# On Scrofula; Naval Surgeons; and Mercurial Inunction in Fevers

**Published:** 1805-07-01

**Authors:** Ralph Cuming

**Affiliations:** Surgeon of H.M.S. Malabar. Yarmouth Roads


### ON SCROFULA; NAVAL SURGEONS; AND MERCURIAL INUNCTION IN FEVERS.

*To the Editors of the Medical and Physical Journal.*

Gentlemen,

MUCH may be done by a little ingenuity in the treatment of this loathsome disease, and particularly in that part of it which relates to the prevention of that deformity, which is generally the result of mal-treament; either in opening these tumours at too advanced a period, or foolishly suffering them to discharge their contents spontaneously. I am fully persuaded that the beauty of many a handsome face, might be most effectually preserved, and those shocking scars, so disgusting to the eye, most perfectly obviated. In delivering my sentiments thus freely to the public, on a subject of the first importance, (fo it is evident that the comfort and happiness of thousands have been sacrificed by the embittering reflection which invariably must originate from conscious deformity, accruing from this insidious disease) it is necessary for me to substantiate the validity of my sentiments, by bringing forward a case or cases exactly in point, which have the firm basis of truth and practice for their support, aided by a theory, as true as the correct solution of any mathematical problem.

A young man, ætatis 20, who had long laboured under scrofula, and who had been under the care of a medical practitioner for some time, made application to me during my residence in Romsey. The parotid and sub-maxillary glands of the left side had suppurated, and left behind a disagreeable cicatrix; the right side was affected in a similar manner, as far as related to the disease, but the integuments preserved their natural colour, for the suppuration tion had not advanced far enough to injure the texture of the cutis vera. In order to prevent this side of the face from being disfigured, I plunged the point of a spear-pointed lancet into the inferior and most depending part of the superior tumour, and discharged its contents by making pressure; the opening was preserved by introducing a small piece of lint, which was removed twice a day. At such dressing, the finger was drawn over the tumor; the matter evacuated, and a piece of adhesive plaster was applied over the lint. One of the sub-maxillary glands was treated in the same manner; and when they were healed, the contrast between the cicatrices on the right and left side was very striking, and of course can only be attributed to a different mode of treatment. When these swellings are suffered to extend the skin to a certain degree, after suppuration has taken place, the ulceration beneath not only becomes more extensive, and consequently more difficult to heal, but retards the cure, produces discolouration of the integuments, and distends them so much, that if not completely destroyed, they are generally puckered in such a manner as to occasion a most unseemly, botched, and what may be termed an unscientific cicatrix. Whereas, by managing these tumors in the way I have described, the parts retain their natural colour, and no mark is left behind, save that of the lancet, which will in a short time become effaced. With respect to the external applications, I conceive nothing can be more serviceable than lint or sticking plaster; all kinds of ointments ought certainly, most religiously to be avoided, as they invariably aggravate this disease, which is entirely of an asthenic diathesis, and therefore requires tonic treatment. Pressure greatly promotes the cure, and will in general succeed, with the assistance of sticking plaster or lint. When there is difficulty in making pressure, astringent, cold, aqueous applications have an excellent effect.

I some time ago was desired to visit a female, whose legs had been for many years covered with scrofulous ulcers, which had gradually got worse and worse; they were considered incurable, and she had been discharged from an infirmary without receiving any benefit. The dressings she used consisted of a variety of greasy preparations, which only served to keep up and increase that atony and irritability which had so long existed. In the course of a few weeks, by firm bandaging with calico wrappers, affusions of cold water, suitable compresses, adhesive strips of plaster and lint, her sores healed up, and she was restored to the use of her legs, which she had for years been deprived of. As for the medical treatment, every one must know, that strong, animal, and gelatinous food is chiefly to be depended on; medicine, when the digestive powers are debilitated, will have a good effect; but it is a difficult thing to produce a speedy change in the solid and fluid parts which constitute the human frame; and which, in all probability is, ab origine, impressed with some properties so peculiar to itself, as in a great measure to enable it to resist, and counteract the intentions of the most skilful physician.

Having, in No. 63 of this Journal, made mention of Naval Surgeons, as a body of men who were not properly remunerated for their services; it is with joy, that I have to communicate the pleasing tidings of His Majesty having most graciously been pleased to take their case into consideration; and by an Order in Council, has caused several regulations to take place relative to their future establishment and emoluments, worthy the King of a great nation; redounding to the honor, and truly indicative of the wisdom of his counsellors; who have at length placed them on that footing, which every liberal and enlightened Naval Officer has long acknowledged to be most justly their due. I am, &c.

*Yarmouth Roads,* RALPH CUMING, M.D.

*May* 31, 1805. Surgeon of H. M. S. Malabar.

P.S. I wish to observe that I have, since joining this ship, experienced farther proofs of the efficacy of mercurial inunctions, in cutting short, and speedily curing typhus fevers; after giving wine, opium, volatile alkali, and other diffusible stimuli, the most patient and fairest trial.

One of my patients took umbrage at what he conceived a very novel treatment, and observed, with an air of great gravity, that he was not *poxed!* But the salutary effects of the remedy, soon convinced him, that it was possessed of more virtue than he was willing to give it credit for.

And those medical gentlemen, who are not contented with that degree of perfection which the medical art has already attained to, will do well to have recourse to a medicine which may truly be considered a sheet anchor, when used with discretion and judgment.

